# Irinotecan—Still an Important Player in Cancer Chemotherapy: A Comprehensive Overview

**DOI:** 10.3390/ijms21144919

**Published:** 2020-07-12

**Authors:** Mateusz Kciuk, Beata Marciniak, Renata Kontek

**Affiliations:** 1 Doctoral School of Exact and Natural Sciences, University of Lodz, 90-237 Lodz, Poland; 2 Department of Molecular Biotechnology and Genetics, Faculty of Biology and Environmental Protection, University of Lodz, 90-237 Lodz, Poland; beata.marciniak@biol.uni.lodz.pl (B.M.); renata.kontek@biol.uni.lodz.pl (R.K.)

**Keywords:** irinotecan, SN-38, topoisomerases, drug combinations, new formulations, drug resistance

## Abstract

Irinotecan has been used in the treatment of various malignancies for many years. Still, the knowledge regarding this drug is expanding. The pharmacogenetics of the drug is the crucial component of response to irinotecan. Furthermore, new formulations of the drug are introduced in order to better deliver the drug and avoid potentially life-threatening side effects. Here, we give a comprehensive overview on irinotecan’s molecular mode of action, metabolism, pharmacogenetics, and toxicity. Moreover, this article features clinically used combinations of the drug with other anticancer agents and introduces novel formulations of drugs (e.g., liposomal formulations, dendrimers, and nanoparticles). It also outlines crucial mechanisms of tumor cells’ resistance to the active metabolite, ethyl-10-hydroxy-camptothecin (SN-38). We are sure that the article will constitute an important source of information for both new researchers in the field of irinotecan chemotherapy and professionals or clinicians who are interested in the topic.

## 1. Introduction

Despite significant progress in medicine, classical chemotherapy still remains the first-line treatment of cancer, especially metastatic tumors. Tumor drug resistance and potential side effects are the main limiting factors in cancer treatment. These factors promote continuous drug development and research that studies the effects of combined treatments of existing drugs. Moreover, the field of drug delivery is expanding rapidly, raising hope for more efficient anticancer therapies [1]. These include the use of liposomal formulations, dendrimers, and nanoparticles as delivery systems. Among conventional antineoplastic drugs, several classes are distinguishable: alkylating agents, antimetabolites, topoisomerase inhibitors, mitotic spindle inhibitors, and others [2,3]. Topoisomerase I inhibitors have been extensively studied since the late 1960s; however, initially, their clinical use was hindered due to their severe toxicity and low stability. Irinotecan as the first member of this drug group was approved for the treatment of cervical, lung, and ovarian cancer in Japan in 1994. In the following years, its use was approved in Europe (1995) and the USA (1996) [4]. More than 20 years later, we aim to summarize what we know about this important anticancer agent and consider the further perspectives of its use.

## 2. Topoisomerases

**Figure 1:**
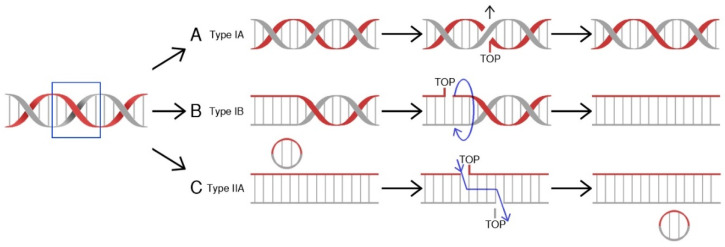
Mechanisms of action for human topoisomerases. (**A**) Topoisomerase IA binds to DNA at a particular binding site, and then claves one strand, forming a transient 5′-phosphotyrosyl bond. The other DNA strand is transferred through the resultant break, allowing DNA to relax. Religation ends the process. (**B**) Toposiomerase IB works in a controlled rotation mechanism and in contrast to type IA enzymes forms a 3′-phosphotyrosyl bond with a DNA molecule. Both type I (A&B) topoisomerases do not require ATP hydrolysis as a source of energy. (**C**) Topoisomerase II binds to both ends of the DNA molecule, forms a double-strand break, and passes a segment of dsDNA through. This reaction is ATP-dependent [19].

Topoisomerases (TOP, Topo) are a class of nuclear enzymes involved in the maintenance of proper DNA topology during replication and transcription [5,6,7,8]. The introduction of single (type I topoisomerases) or double-strand breaks (type II topoisomerases) allows supercoiled DNA to relax and effectively serve as a template [7,9,10]. Structurally, type I topoisomerases are monomeric proteins, while type II topoisomerases work as homodimers. Topoisomerases catalyze a controlled and reversible transestrification reaction in which a tyrosyl group at the active center of the enzyme is linked with a phosphor group of the DNA strand. Type IA enzymes covalently attach tyrosine (Y723) at the active center to the 5′-phosphoryl group of DNA and work in a mechanism called “strand passage” [11]. In contrast, IB and IC topoisomerases attach tyrosine to a 3′-phosphoryl group of DNA molecule in a “controlled rotation” or “swivel” mechanism [12,13,14]. On the other hand, type II enzymes bind both ends of the target DNA molecule. This reaction is followed by DNA break formation. Type II topoisomerases introduce double-strand breaks and pass another DNA duplex through formed breaks [15,16], while in the action mechanism of topoisomerases I, the other DNA strand is transferred through the aforesaid, resultant break before the religation process [17]. The arisen covalent bond is hydrolyzed immediately after relaxation of the DNA molecule and the cut strand is being resealed (Figure 1). The energy required for the “controlled rotation” catalysis carried out by topoisomerases I is obtained from the helical torsions of the DNA template (as a result of intrinsic strain energy) [17], while type II topoisomerases require ATP hydrolysis as a source of energy [15]. The stabilization of temporary introduced strand breaks has been proven to be an effective therapeutic strategy used to treat cancer [18].

## 3. Topoisomerase Inhibitors

Topoisomerase inhibitors are compounds that have been widely used in cancer treatment. There are several molecular mechanisms that can be used to inhibit topoisomerase action. The first one is based on substrate competitiveness. An inhibitory compound directly binds to the active site of the enzyme, preventing native substrate binding. So far, no such compounds have been identified. The second one is based on interactions between the protein, DNA, and topoisomerase inhibitor, which is suitably called “topoisomerase poison”. The inhibition results in DNA religation blockage and cleavage complexes formation [20]. The last possible mechanism depends on ATP-hydrolysis prevention. This inhibition is achieved through the binding of a small molecular inhibitor to the ATP binding site of DNA topoisomerases [5,21,22]. The commonly used topoisomerase I inhibitors with their properties are summarized in Table 1.

## 4. Irinotecan (CPT-11)

**Figure 2:**
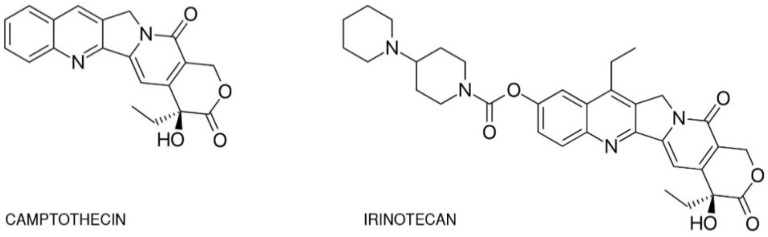
Chemical structures of camptothecin and irinotecan.

One of the best-studied topoisomerase I inhibitors is irinotecan. Since its introduction in 1996, 7-ethyl-10-[4-(1-piperidino)-1-piperidino]-carbonyloxycamptothecine (CPT-11) has been used to treat various types of cancer such as: pulmonary [23,24,25,26], pancreatic, gastric [27,28], ovarian [29,30], cervical [31], colorectal [32], and others [33]. Isolated from the Chinese tree *Camptotheca acuminata*, camptothecin alkaloid was the base for semi-synthetic, water-soluble anologs such as irinotecan (CPT-11, drug name: Camptosar^®^, Campto^®^) and topotecan (Hycamtin^®^) that have been approved by the US Federal Drug Administration for clinical use (in 1996 and 2007 respectively) [19]. Irinotecan is a pentacyclic alkaloid provided with a bis-piperidine side chain, which contributes to its water solubility (Figure 2) [34]. Similar to other campthothecins, irinotecan undergoes structural changes depending on the physiological pH of the cellular environment. Irinotecan is considered to be active in lactone structural form but impermeable through cell membranes and therefore inactive in carboxylate form [35,36].

## 5. Mechanism of Action

**Figure 3:**
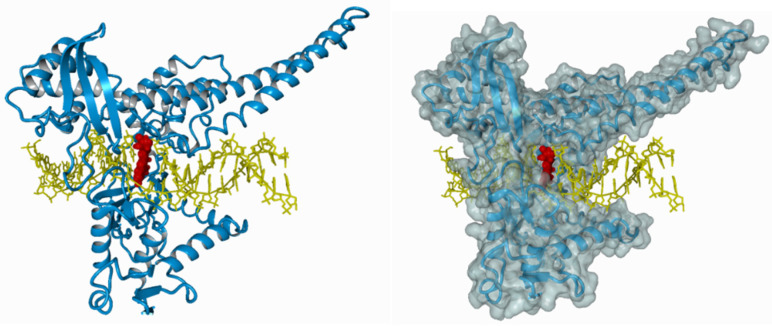
The ternary irinotecan–topoisomerase I–nicked DNA complex. Inhibition of topoisomerase I (blue) bound to the DNA molecule (yellow) with CPT-11 (red).

The ternary irinotecan–topoisomerase I–nicked DNA complex (Figure 3) disables the religation of the nicked strand and prevents topoisomerase release. Collision of the formed complex with advancing replication forks results in lethal double-strand break (DSB) formation [37]. This contributes to DNA damage checkpoint signalling, replication fork arrest, and cell death [38]. The ATM–CHK2–TP53 is the main signaling transduction pathway activated in cells that accumulate DSB in response to CPT-11 treatment [39]. The uniqueness of topoisomerase I inhibitors is manifested by their dose-dependent increase in enzyme inhibition with a rise in cellular topoisomerase concentration. Therefore, the sensitivity of cells to topoisomerase inhibitors mainly depends on the concentration of topoisomerase inside the cell. Cancer cells express higher yields of the enzyme and thus are more prone to topoisomerase poisons. This is particularly observed in colon, esophageal, and cervical cancers, as well as non-Hodgkin’s lymphoma. Moreover, it was shown that the expression of topoisomerase I may be 14–16 times higher in cancer cells than in normal cells surrounding the tumor [40,41].

It has been proven that irinotecan acts as an efficient radiosensitizer in vitro. The radiosensitizing effects of irinotecan are attributed to ATM/CHK/CDC25C/CDC2 signaling, which leads to G2/M phase arrest and the apoptosis of colorectal cancer cells [42].

**Figure 4:**
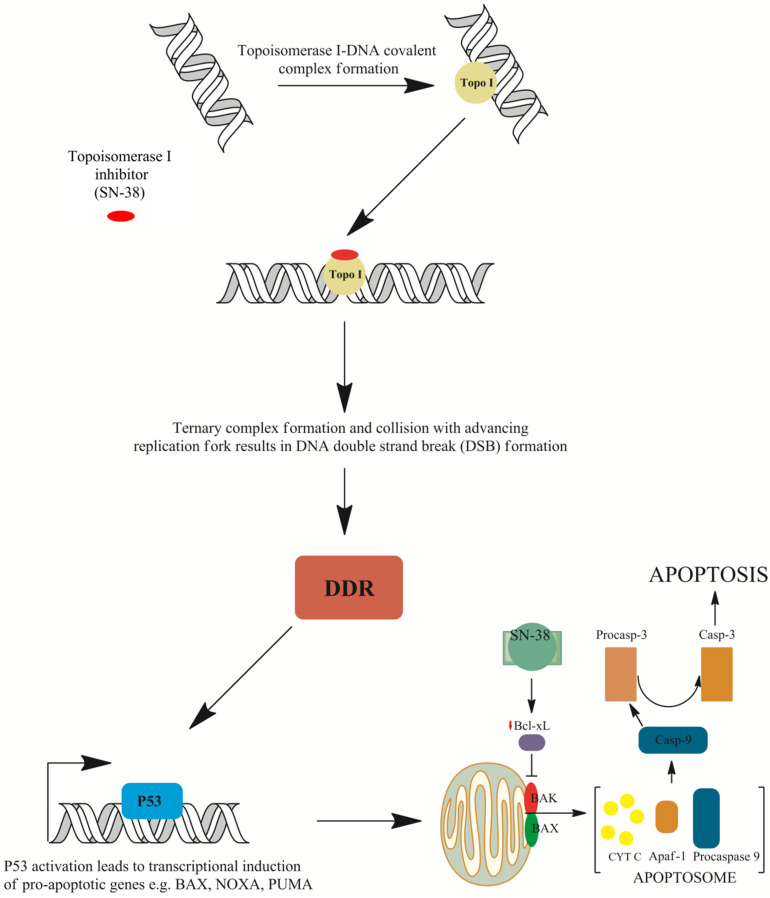
Mechanisms of cell death induced by irinotecan. The ternary irinotecan–topoisomerase I–nicked DNA complex with topoisomerase I (Topo I) inhibitor (SN-38) disables religation of the nicked strand and prevents topoisomerase release. Collision of the formed complex with advancing replication forks results in the formation of double-strand breaks (DSBs). This is followed by DNA damage response (DDR) signaling and TP53-induced gene expression of pro-apoptotic genes such as apoptosis regulator (BAX), phorbol-12-myristate-13-acetate-induced protein 1 (NOXA), or p53 up-regulated modulator of apoptosis (PUMA). Furthermore, down-regulation of the Bcl-2-like protein 1 isoform protein (BCL-xL) protein allows BAX and Bcl-2 homologous antagonist (BAK) embedding into the mitochondrial membrane and the formation of pores that allow the release of cytochrome C (cytC) from mitochondria and the subsequent formation of apoptosome with apoptotic protease-activating factor 1 (APAF-1) protein and procaspase-9. The activation of procaspase to caspase 9 (Casp-9) results in proteolytic cleavage and the conversion of procaspase 3 into active caspase 3 (Casp-3), which executes apoptosis [37,38,44,49,51].

However, recent studies suggest that topoisomerase I may not be the only target of irinotecan. The active metabolite of irinotecan, ethyl-10-hydroxy-camptothecin (SN38), interacts with the mouse double minute 2 homolog (MDM2) protein involved in TP53-mediated cell death and anti-apoptotic Bcl-2-like protein 1 isoform protein (BCL-xL) [43]. SN-38 was also shown to induce both cellular tumor antigen p53 (*TP53*) expression and phosphorylation, which is accompanied with a concomitant elevated expression of downstream apoptosis-inducing proteins such as apoptosis regulator (BAX), caspase-3, and caspase-9 in human hepatocellular carcinoma cells in vitro. This was accompanied with the diminished expression of BCL-xL and proved that SN-38 induces cell apoptosis in a TP53-dependent mechanism (Figure 4) [44]. McDermott et al. [45] reported that the apoptotic response to irinotecan can be in fact tumor necrosis factor receptor superfamily member 6 (FAS)-mediated and may not rely on TP53 activation solely. In addition, pro-apoptotic gene expression was altered in acute myeloid leukemia cells exposed to SN-38. Anti-apoptotic BCL2 expression was seen to be down-regulated, while an increase in B-cell lymphoma/leukemia 10 (BCL10), a protein responsible for caspase recruitment, was shown to be up-regulated and was established as an important contributor to cell apoptosis [46]. Moreover, cyclin dependent kinase 4 (CDK4) expression was slightly and temporary down-regulated, advocating the G1-S transition block through the diminished phosphorylation of retinoblastoma gene product (pRb). Gene expression altered in response to irinotecan may be highly complex, as it was shown by Minderman et al. [46]. Furthermore, it was demonstrated that mitogen-activated serine/threonine protein kinases (MAPKs) may play an important role in irinotecan-induced apoptosis. One of the MAPKs that has a dualistic role in cell survival is p38. Long-term p38 activation is thought to fire up pro-apoptotic signaling. On the other hand, it was shown that colon cancer cell survival is dependent on p38, and its inhibition leads cell cycle arrest and apoptosis. Rudolf et al. [47] demonstrated that CPT-11 treatment may activate p38 signaling in cells and caspase-mediated apoptosis in a dose-dependent manner. However, they implicated that lower doses of topoisomerase poison activate p38 protein, but only in a certain fraction of cells. Thus, one can assume that the role of p38 in apoptotic response may be context dependent and remains to be elucidated.

Various genes were reported to be involved in the apoptotic response to irinotecan [48]. However, apoptosis is not the only mechanism that is thought to be triggered in response to irinotecan treatment. Instead of apoptosis, cells may undergo senescence. These feedback mechanisms may be mediated through RAC–alpha serine/threonine–protein kinase (AKT) signaling. Inhibiting senescence with p21^waf1^ inhibitory compounds leads to apoptosis activation mainly through phorbol-12-myristate-13-acetate-induced protein 1 (NOXA) up-regulated transcription and induced myeloid leukemia cell differentiation protein (MCL-1) inactivation (Figure 4) [49]. This is concurrent with findings obtained by Was et al. [50]. The team treated human colon cancer HCT116 and SW480 cells with 5-fluorouracil, oxaliplatin, and irinotecan. Cells exposed to 5-fluorouracil (5-FU) or irinotecan showed several hallmarks of stress-induced premature senescence (SIPS): growth arrest, increase in cell size and granularity, polyploidization of cells, elevated activity of the senescence associated β-galactosidase, accumulation of P21 and cyclin D1 proteins, and the senescence-associated secretory phenotype. Moreover, they found that a subpopulation of senescent colon cancer was present, and these cells exhibited features characteristic for stem cells: elevated expression of homeobox protein NANOG and signal transducer CD24. These findings indicate that senescence may be the second mechanism of death induced by SN-38, and it may be responsible for resistance to chemotherapy and tumor recurrence [50].

A recent study of Bao et al. [52] indicates that the mechanism of irinotecan/SN-38 action may also depend on cell type. In cells with a quiescent proliferating rate but rich in mitochondria such as hepatocytes, drug action relies on the induction of mitochondrial dysfunction and oxidative stress. In highly proliferative cells—for example, cancer cells, but also normal bone marrow or intestinal basal cells—the main target of irinotecan/SN-38 action is the inhibition of topoisomerase I. A comparative study of cell metabolic signature showed that in the presence of irinotecan/SN-38, cancer cells (MDA-MB-231 and T47D) accumulated mostly pyrimidine/purine nucleosides and nucleobases in culture medium, which are probably consequence of DNA damage and subsequent DNA degradation. At the same time, hepatocytes (HepatoCells) accumulate amino acid metabolites and acylcarnitines, which may indicate that there is some mitochondria dysfunction [52]. The possible roles, molecular targets, actions, and consequences of irinotecan application are summarized in Table 1.

## 6. Metabolism, Pharmacogenetics, and Toxicity

**Figure 5:**
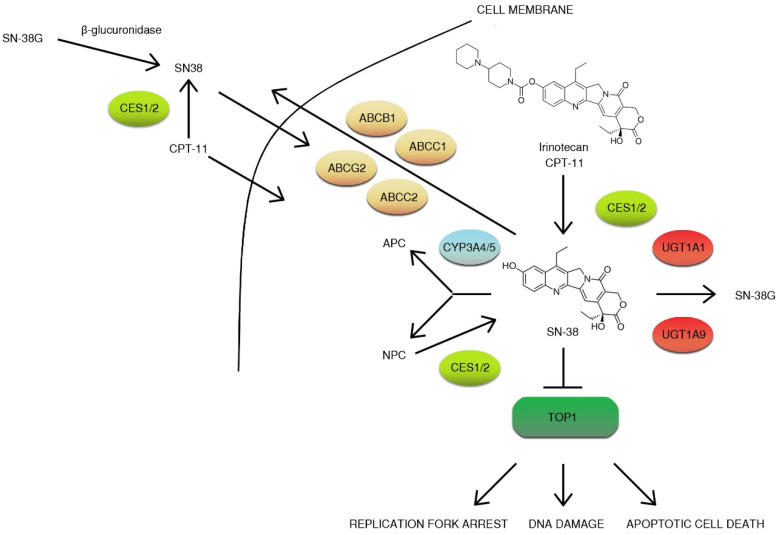
Overview of irinotecan metabolism. CPT-11 is a prodrug that is converted to active metabolite ethyl-10-hydroxy-camptothecin (SN-38) by liver carboxylesterase converting enzymes (CES1/2) and is then transported to the liver by 1B1 polypeptide (OATP1B1) and inactivated by microsomal uridine 5′-diphospho-glucuronosyltransferase enzymes (UGTs): UGT1A1 and UGT1A9. Irinotecan is transported to bile by a group of the ATP-binding cassette transporters (ABC transporters): ABCB1, ABCC1, ABCC2, and ABCG2. Irinotecan is efficiently metabolized by cytochrome P450 enzymes: CYP3A4 and CYP3A5. This results in the generation of less active metabolites APC (7-ethyl-10-[4-N-(5-aminopentanoic acid)-1-piperidino] carbonyloxycamptothecin) and NPC (7-ethyl-10-[4-amino-1-piperidino] carbonyloxycamptothecin). NPC (but not APC) can be further converted to SN-38 by CES1, and CES2 gut microbiota may also participate in irinotecan metabolism by the production of β-glucuronidase, which catalyzes the breakdown of SN-38G to SN-38. The inhibition of topoisomerase I (TOP1) results in DNA damage, replication fork arrest, and apoptotic cell death [53,54,55,56,57,58,59,60,61,62].

CPT-11 is a prodrug that is converted to active metabolite ethyl-10-hydroxy-camptothecin (SN-38) by liver carboxylesterase converting enzymes (CES1/2) and butyrylcholinesterase (hBChE) in blood plasma [53]. Then, SN-38 is then transported to the liver by the 1B1 polypeptide (OATP1B1) and inactivated by microsomal uridine 5′-diphospho-glucuronosyltransferase enzymes (UGT): UGT1A1 and UGT1A9 [54,55,56]. Other non-hepatic UGT enzymes (UGT1A1, UGT1A7, UGT1A10) convert SN-38 to glucuronide (β-glucuronide conjugate) (SN-38G) secreted with bile [57]. Glucuronidation greatly increases the polarity of SN-38, promoting the elimination of drug from the body [58]. Irinotecan is transported to bile by a group of the ATP-binding cassette transporters (ABC transporters): ABCB1, ABCC1, ABCC2, and ABCG2 [59]. The metabolism of the drug is complex. Irinotecan is efficiently metabolized by cytochrome P450 enzymes: CYP3A4 and CYP3A5. This results in the generation of less active metabolites APC (7-ethyl-10-[4-N-(5-aminopentanoic acid)-1-piperidino] carbonyloxycamptothecin) and NPC (7-ethyl-10-(4-amino-1-piperidino] carbonyloxycamptothecin). NPC (but not APC) can be further converted to SN-38 by CES1 and CES 2 [60,61,62]. Gut microbiota may also participate in irinotecan metabolism by the production of β-glucuronidase that catalyze the breakdown of SN-38G to SN-38. Irinotecan metabolites, except for the glucuronide form of SN-38, are eliminated with feces (Figure 5) [63]. Irinotecan elimination rates are highly variable with several factors affecting drug clearance. Irinotecan pharmacokinetics can be altered by age, sex, dose, administration timing, or hepatic function [48,64]. Moreover, some of the mentioned factors were reported to have an impact on toxicity. Neutropenia, one of the most commonly faced side effects (18–54% patients), is more frequently present in females on FOLFIRI (5-fluorouracil, leucovorin and irinotecan) regimen than males [64].

As mentioned, neutropenia and severe, delayed diarrhea are the major symptoms of respectively hematologic and gastrointestinal toxicity that limit irinotecan treatment. Many cases indicate that irinotecan treatment may also lead to the development of steatohepatitis [52,65]. Bao et al., 2019 attempted an early detection of both antitumor activity and side effects to irinotecan based on metabolic changes in patients’ plasma after drug administration. They observed a time-dependent accumulation of metabolites such as acylcarnitines and specific amino acids, which may provide evidence about mitochondrial dysfunction and oxidative stress in the liver. However, the level of circulating nucleobases in patients’ plasma may reflect the nucleotide degradation in cancer cells and highly proliferative normal cells [52].

A large body of evidence indicates that irinotecan-treated patients may suffer from cholinergic-like symptoms: bradycardia, sweating, lacrimation, abdominal pain, and diarrhea. The last one may occur immediately after drug infusion (early-onset diarrhoea) or 8 to 10 days after drug administration (late-onset diarrhoea) and represents a major burden in irinotecan-based chemotherapy [66,67].

## 7. Most Important Single-Nucleotide Polymorphisms (SNPs) Associated with Irinotecan Use

Genetic polymorphism in genes encoding metabolic enzymes is the key factor that contributes to drug conversion, toxicity, and elimination [68]. Irinotecan toxicity profoundly depends on the glucuronidation rate; thus, certain polymorphisms in the UDP glucuronsyltransferase gene (UGT1A1) are associated with a higher risk of severe toxicity of SN-38 in patients [69]. The gene product is usually defective and exhibits reduced glucuronidation activity, affecting the drug metabolism. UGT1A1*6 and UGT1A1*28 polymorphisms are established as the most significant in the prediction of adverse drug reactions (ADR) occurrence such as neutropenia and diarrhea. Meta-analysis performed by Yang et al. [70] indicates that irinotecan-induced toxicity correlates with UGT1A1 polymorphisms and is associated with drug dosing in certain cancer types. Genotyping for variants of genes responsible for irinotecan efflux may by crucial for dosing optimization and useful for personalized treatment [70].

ABCB1, ABCC1, ABCC2, and ABCG2 efflux transporters have a key role in the time of exposure to irinotecan and its metabolites [59,71,72]. Genetic polymorphism in aforementioned genes may alter the expression of transporter proteins and affect the drug metabolite disposition. It was shown that SNPs in the ABCB1 gene (2677TT and 3435TT) are connected with higher cellular efflux activity in patients with advanced non-small cell lung cancer (NSCLC) [71]. There is also evidence that genetic variants of ABCB1 and ABCC2 are relevant to irinotecan toxicity [59,71,72]. In the case of ABCG2 gene polymorphisms (rs2231142, 421C>A), contradictory findings are available [72,73].

Other members of the drug transporting system responsible for irinotecan influx from blood to hepatocytes such as SLCO1B1 may be involved in the occurrence of severe toxicity to irinotecan. The presence of a genetic variant of SLCO1B1 (521T > C) was correlated with higher toxicity in metastatic colorectal cancer patients treated with irinotecan as the first line of treatment [74].

A genome-wide association study evaluated certain polymorphisms that can help identify patients with neutropenia and diarrhea hazards after irinotecan treatment. Forty-nine SNPs associated with the risk of diarrhea and 32 SNPs related to the risk of neutropenia have been identified. Among the polymorphisms associated with the risk of severe grade III diarrhea, 3 novel SNPs were identified: C8orf34 rs1517114, FLJ41856 rs1661167, and PLCB1 rs2745761. PDZRN3 rs11128347, SEMA3C rs11979430, and rs7779029 genetic variants were among the selected candidates responsible for the occurrence of grade IV neutropenia after irinotecan treatment [75]. The UGT1A1*93 polymorphism also seems to be potentially significant for the occurrence of neutropenia following irinotecan treatment [59].

## 8. Irinotecan Anitcancer–Drug Combinations

Many anticancer agents have been used in combination with irinotecan. The sequential therapy of topoisomerse II inhibitors followed by irinotecan or the simultaneous administration of the drugs was tested in preclinical trials. However, this combinatory approach was rejected due to severe neutropenia after the sequential use of drugs [76]. Irinotecan has been used in synergy with antimetabolites such as 5-fluorouracil (5-FU) in gastrointestinal malignancies [33]. 5-FU inhibits thymidylate synthase activity and disables thymidine monophosphate production (TMP). Low levels of TMP led to the disruption of DNA replication and resulted in the inhibition of cell proliferation. Moreover, 5-fluorodeoxyuridylate, an activated 5-FU that is incorporated during DNA and RNA synthesis, contributed to DNA damage [77]. A combination of 5-FU and irinotecan has received considerable attention due to additive or synergistic effects [78]. The administration of irinotecan prior to 5-FU enhanced the cytotoxicity of the second drug by the reduction of thymidylate synthase expression. However, several studies suggest that the increased cytotoxicity may result from the independent action of either drug alone [33]. Furthermore, a combination of irinotecan with 5-FU and biomodulatory compounds such as leucovorin (FOLFIRI regimen) increased the effectiveness of colon cancer treatment. Saltz et al. [79] reported that the ternary treatment scheme was superior to either irinotecan monotheraphy or 5-FU and leucovorin with significantly longer overall or progression-free survival and higher response rates. Similar results were obtained by Douillard et al., but the irinotecan arm was not included in the experimental design [79]. FOLFIRI is currently evaluated as a third-line therapy in patients with metastatic gastric cancer and as a second-line treatment of metastatic biliary tract cancer [80,81]. The FOLFIRI regiment was later improved with another cytotoxic drug, oxaliplatin, to two similar schedules of FOLFOXIRI and FOLFIRINOX (higher dose of irinotecan and 5-FU in bolus). The FOLFOXIRI regimen was shown to increase treatment efficiency in colorectal cancer, while FOLFIRINOX was shown to be an effective combination for the treatment of advanced pancreatic cancer [82]. FOLFIRI and FOLFOXIRI regimens were further modified with the addition of monoclonal antibodies—bevacizumab [83], cetuximab [84], and panitumumab [64,85].

Among the alternative therapies, platinum-based anticancer drugs such as cisplatin or oxaliplatin have been employed in combination with irinotecan. These have been proven to be effective strategies to treat malignancies [86,87,88]. Cisplatin and oxaliplatin are known bifunctional alkylators that react with adjacent guanine residues creating inter or intra-strand crosslinks, interfering with DNA replication or transcription, and contributing to cell death [89]. Various analogs of these drugs were tested in vitro. Kontek et al. reported a significant increase in the genotoxic potential of irinotecan when the drug was combined with platinum compound-trans-[PtCl2(4-pmOpe)2] and tested in gastrointestinal and pulmonary cancer cells [90]. Extensive studies were conducted in order to compare different combinations of irionotecan, cisplatin, and etoposide in the treatment of NSCLC [91] and small cell lung cancer (SCLC) [92,93,94]. Another commonly used anticancer agent paclitaxel (microtuble disrupting drug) combined with irinotecan exhibited synergistic anticancer activity and showed an increase in SN-38 exposure [95].

Tyrosine kinase inhibitors represent another class of compounds that have been used in combination with irinotecan. Protein tyrosine kinases (PTKs) belong to a family of related proteins that have been shown to play major role in cancer development. They can be subdivided into receptor tyrosine kinases embedded in the phospholipid bilayer and non-receptor kinases. Ligand binding results in receptor dimerization and self-cross-transphosphorylation that leads to initiation of the signaling pathway. On the other hand, non-receptor kinases are cytosolic proteins that phosphorylate downstream effector proteins [96]. Combinations of irinotecan with tyrosine kinase inhibitors—apatinib [97], dasatinib [98], lapatinib [99], pazopanib [100], regorafenib [101] and sunitinib [102]—have been recently tested with different efficacies and endpoints.

More recently, Reita et al. [103] showed a synergistic antitumor activity of serine/threonine-protein kinase mTOR inhibitors (AZD8055, AZD2014) and irinotecan in vitro and in vivo. The combination of drugs reduced colon cancer cell lines motility by 70% compared to 40% reduction by AZD2014 alone. Interestingly, the combination but not drugs alone reduced cell invasion to a level of 70%. Furthermore, in vivo studies revealed a significant reduction of ectopic patient-derived colon tumor growth when a combination of drugs was applied. The mentioned combination was proven to be more effective than commonly used regimens FOLFOX (leucovorin, 5-FU, and oxaliplatin) and FOLFIRI. Moreover, the use of mTOR inhibitors and irinotecan completely inhibited lung and liver metastases induced from the implantation of SW480 cells [103].

## 9. New Irinotecan Formulations

Many different approaches were taken to overcome the problem of bioavailability of the drug or its active metabolite SN-38. These include the design of liposomal formulations, nanoparticles, polymer conjugates, dendrimers, peptides, or carbohydrates [104].

Liposomal irinotecan (nal-IRI, ONIVYDE) was approved in 2015 [105]. Since then, new liposomal irinotecan formulations have been developed and used as second-line treatments of metastatic pancreatic cancer [106,107]. Liposomes are phospholipid bilayers equipped with inner aqueous pockets that are used as drug delivery enhancers of hydrophobic and hydrophilic agents [108]. Liposomes provide a protective layer that shelters encapsulated drug from the structural alterations or chemical degradation [109]. Furthermore, the covalent adherence of polyethylene glycol (PEG) molecules can be used to improve the systemic circulation of drugs [110]. PEGylated liposomal formulation of irinotecan (MM-398) improved the cytotoxic effects of irinotecan in a mouse model of brain metastasis compared to irinotecan monotherapy [111]. Liposomal formulations of irinotecan are promising new cytotoxic agents that can be utilized to treat other malignancies such as metastatic breast cancer [105]. Zhang et al. [112] conjugated irinotecan with a series of fatty acids to increase its lipophilicity and allow particles to self-assemble in an aqueous environment in order to protect estrified irinotecan from bond hydrolysis by carboxylesterases. This approach resulted in higher intracellular accumulation and an elevated cytotoxicity of irinotecan [112].

One of the main factors that favored liposomal drug formation was the asset of alleviated toxicity due to more directed delivery. Different kinds of carriers have been used to achieve this goal. However, most of them fail to serve their purpose in vivo, while exhibiting a high potential in vitro.

Not only liposomal formulations but also graphene-based irinotecan formulations were prepared to increase the effectiveness of the drug. Graphene with a sp2-hybridized 2D framework has attracted much attention from scientists because of its outstanding properties. Graphene oxides (GOs) provide a relatively high surface for loading of drug. Moreover, the oxidation of graphene results in the formation of chemical groups such as such hydroxyl (–OH), epoxy (> O), and carboxylic (–COOH) groups that can be modified according to the purpose. Furthermore, graphene has high membrane penetrating potential, and the formulations of drugs can be easily uptaken by the cells. Karki et al. [113] performed research on SN-38 loaded on graphene oxides (GOs) modified with either polyvinylpyrrolidone (PVP) or excipient β-cyclodextrin (β-CD). The team analyzed the release of the drug from the nanocarriers and assessed their cytotoxicity in human breast cancer cells (MCF-7). The researchers showed that the SN-38 loaded on nanocarriers exhibited higher cytotoxic potential in the tested cell line. Moreover, the GO–PVP nanocarrier had higher cytotoxic activity than the GO–β-CD nanocarrier, indicating that GO–PVP is a more effective drug delivery system [113].

Alibolandi et al. [114] developed a PEGylated acetylated carboxymethylcellulose conjugate of SN38 and covalently attached it to an aptamer against CD133 to ensure the selective delivery of the drug to colorectal cancer stem cells. This approach allowed the nanoparticles to be uptaken by a CD133-expressing HT29 cell line in vitro. Furthermore, the use of nanoconjugates resulted in an enhanced cytotoxicity of the drug compared to the non-targeted self-assembled nanoconjugate [114].

Valencia et al. proposed a combinatory therapy of both cisplatin and irinotecan encapsulated in poly(d,l-lactide-*co*-glycolide)-*co*-poly(ethylene glycol) (PLGA–PEG)-based nanoparticles. The self-assembly of molecules allowed irinotecan to be passively incorporated into the complexes. The formed nanoparticles were directed toward prostate cancer cells overexpressing prostate-specific membrane antigen (PSMA) receptors, by using the PSMA ligand S,S-2-(3-[5-amino-1-carboxypentyl]-ureido)pentanedioic acid. This resulted in selective endocytotic uptake and the controlled release of drug, allowing complexes to act as cytotoxic agents. Both agents exhibited synergistic activities, resulting in elevated cell killing [115].

On the other hand, Onishi et al. [116] examined irinotecan nanoparticles with poly(ethylene glycol)-block-poly(propylene glycol)-block-poly(ethylene glycol) for their antitumor potential in mouse sarcoma model in vivo. The group suggested that the tested nanoparticles may exhibit cytotoxic potential in solid tumors distant from the administration site [116].

Machmoudi et al. [117] constructed PEGylated polyamidoamine (PAMAM) dendrimers containing SN-38 conjugated with peptides: BR2 and CyLoP1. The team assessed both the cytotoxicity and uptake of the formulation by the murine colon carcinoma (C26) cell line. In vitro studies showed that the formulation was much more cytotoxic (range of 154.4-635 nM) compared to SN38 in native form. In addition, the dendrimers were uptaken more promptly by cells compared to SN38. Furthermore, in vivo studies were carried out in order to estimate antitumor efficacy. The results showed that the new formulation enhanced drug accumulation efficiency at the tumor site and exhibited higher anti-tumorigenic efficacy compared to SN-38 alone [117].

Wang et al. developed novel nanoparticles that consisted of hyaluronic acid, poly(lactic-co-glycolic acid), chitosan, and pluronic F-127 as a nanocarrier of irinotecan and doxorubicin [118]. Recently, Hyaluronic Acid ChemoTransport (HyACT^®^) has been employed as a carriage system of irinotecan. Preclinical studies using the mentioned formulation showed improved responsiveness in CD44 positive tumor cells. In addition, it has been demonstrated that the combination resulted in improved progression-free survival in metastatic colorectal cancer when compared to normal irinotecan monotherapy [119].

Interesting studies were carried by Naumann et al. [120]. The team employed SN-38 conjugated to gold nanoparticles via oligonucleotides complementary to specific mRNAs unique to the cancer cells of Ewing sarcoma. In a cancer cell, the SN38-conjugate oligonucleotide is released, allowing the inhibition of topoisomerase by SN-38. The obtained result showed that the drug was efficiently delivered and selectively released in both in vitro and in vivo conditions [120].

Zashikhina et al. [121] developed self-assembled poly(l-lysine)-b-poly(l-leucine) (PLys-b-PLeu) polymersomes. The cytotoxicity of polypeptide polymerosomes was tested on three cell lines: HEK, NIH-3T3, and A549. The researchers found that the carriers did not exhibit any cytotoxic activity in the tested cell lines. Moreover, the loading of irinotecan into polymersomes resulted in similar antitumor activity in vitro to that observed for free drug [121]. The summary of new drug formulations is provided in Table 2 below.

## 10. Mechanisms of Tumor Cells Resistance

Many complex mechanisms cooperate in the development of drug resistance in cancer cells. The active efflux of chemotherapeutic agents by proteins from the ATP-binding cassette (ABC) family is one of the best known. The overexpression of *ABCG2* is associated with multi-drug resistance in many human cancer cells. In addition, in the case of irinotecan resistance, a predictive role of ABCG2 drug transporter protein was evaluated, but unfortunately, contradictory results have been obtained [122].

Differences in the chemosensitivity of patients to irinotecan treatment may also be caused by alterations in drug metabolism among patients. In this context, carboxylesterase 2 (CES-2) is the main enzyme responsible for irinotecan activation and thus may play a key role in drug resistance. Shaojun et al., based on an immunohistochemical analysis of the CES-2 marker, observed a correlation between the curative effect of irinotecan and the expression of CES-2 in metastatic colorectal cancer (mCRC) patient samples [123]. As irinotecan/SN-38 is a topoisomerase I inhibitor, the expression of this enzyme was also evaluated as a possible drug-response indicator. Two large clinical studies in patients with CRC were conducted: CAIRO (545 patients involved) [124] and MRC FOCUS (1313 patients involved) [125]. Interestingly, these randomized trials brought contradictory findings. In the MRC FOCUS studies, medium and high levels of Topo-1 expression were positively correlated with the response to irinotecan, while in the CAIRO study, such correlation was not observed [126]. Subsequently, Shaojun et al. (in research comprising 98 patients) showed that Topo-1 expression is associated with PFS (progression-free survival) and OS (overall survival) in mCRC patients but indicated that other molecules may be involved [123].

Another possible hypothesis assumes that new topoisomerase I mutations (p.R621H, p.L617I, and p.E710G) and their localization established by Gongora et al. may play a prominent role in the interaction between TOP1 and SN-38 as they enhance the linkage flexibility [127]. However, these mutations were detected only in vitro in SN-38-resistant HCT116 sublines. In fact, examinations on colorectal cancer patient’s samples have not demonstrated the presence of these mutations [127].

Proteomic analysis conducted by Peng et al. [128] indicates that the 15 proteins involved in mechanisms such as metabolism, apoptosis, cellular transcription, differentiation, proliferation, and many others could be up- or down-regulated in resistance process. For example, in selected irinotecan-resistant human colon adenocarcinoma LoVo cells (LoVo/irinotecan), anti-apoptotic Cofilin 1 was overexpressed, which is in agreement with data obtained for other multi-drug resistant (MDR) human pancreatic cancer sublines such as EPP85-181RDB or EPP85-181RNOV [128]. Paillas et al. [129] in turn indicate a role of α and β isoforms of p38 kinase in cancer resistance prediction. A study carried out on HCT116-resistant cells, xenograft models, and clinical samples from colorectal cancer patients showed that the activation (phosphorylation status) of p38 may contribute to irinotecan/SN-38 resistance [129].

Obtained data suggest that not one, but many different mechanisms and signaling pathways may act simultaneously or complementarily in the development of drug resistance. Plenty of promising in vitro studies have been conducted, but most of them fail the clinical implementation or simply do not confirm results obtained from the previous studies.

## 11. Conclusions

Irinotecan has been used in the treatment of various malignancies for many years. However, the therapeutic use of irinotecan and its active metabolite, SN38, is limited by its hydrophobicity, low stability at physiologic pH, and side effects. These obstacles can be overcome by new drug formulations. The use of irinotecan/SN38 containing nanoparticles, polymer conjugates, dendrimers, peptides, and carbohydrates significantly improves the clinical utility of the drug. Furthermore, understanding individual genetic background is crucial for implementation of the treatment and management of potentially life-threatening side effects. The genetic profiling of patients may provide useful information for clinicians. The identification of the genetic polymorphisms in genes involved in metabolism is of particular importance, as they can influence the use of the drug alone and in combination with other anticancer agents. Moreover, with the use of nanocarriers, both drugs can be delivered simultaneously or in the same vehicle to ensure their delivery to target cells. Progress in the understanding of irinotecan-mediated cell death may lead to the better management of resistance to treatment and lower the chance of cancer remission in the future.

## Figures and Tables

**Table 1:** Possible roles, molecular targets, actions, and consequences of irinotecan/SN38 application provided with references. ATM: Serine-protein kinase ATM, CDC2: cyclin-dependent kinase 1, CDC25C: M-phase inducer phosphatase 3, CHK: serine/threonine-protein kinase CHK, DSB: double-strand break, FAS: tumor necrosis factor receptor superfamily member 6, MAPK: mitogen-activated serine/threonine protein kinase, MDM2: mouse double minute 2 homolog, p38: mitogen-activated protein kinase p38, Top I: topoisomerase I, TP53: cellular tumor antigen p53.

Irinotecan/SN38				
Role	Molecular Target	Action	Consequence	References
inhibitor	Top I	Stabilization of Top I–DNA complex	replication fork arrest DSB formation cell death	[37,38,46]
radiosensitizer (in vitro)	ATM/CHK/CDC25C/CDC2 pathway	Increase of gene expression/ activation of DNA damage response	G2/M phase arrest apoptosis	[42]
inhibitor	MDM2	TP-53-mediated gene expression induction	TP53 release G2/M phase arrest apoptosis	[43]
inductor (in vitro)	TP53	Induction of gene expression	increase expression of: BAX, caspase-3 and caspase-9 apoptosis	[44]
inductor	FAS	Up-regulation of FAS expression in a TP53-independent mechanism	cell death by DISC	[45]
activator	p38	Activation of MAPK signaling pathway	cell cycle arrest apoptosis	[47]

**Table 2:** New irinotecan formulations with effects of modifications and references.

New Formulation	Effect of Modification	Reference
PEGylated liposomal irinotecan	Improved cytotoxic effects of irinotecan in mouse model of brain metastasis compared to irinotecan monotherapy.	[111]
Irinotecan (Iri)-fatty acid prodrugs (Iri5C, Iri-8C, and Iri-12C) with alkyl chains of different lengths synthesized by esterification using DCC (dicyclohexylcarbodiimide) and DMAP (4-dimethylamino-pyridine).	Higher intracellular accumulation of the drug and elevated cytotoxicity of irinotecan.	[112]
SN-38 loaded on graphene oxides (GOs) modified with either polyvinylpyrrolidone (PVP) or excipient β-cyclodextrin (β-CD).	SN-38 loaded on nanocarriers exhibited higher cytotoxic potential in the MCF-7 cell line. The GO–PVP nanocarrier had higher cytotoxic activity than the GO-β-CD nanocarrier, indicating that the GO–PVP nanocarrier is a more effective drug delivery system.	[113]
PEGylated acetylated carboxymethylcellulose conjugate of SN38 covalently attached it to an aptamer against CD133.	Enhanced uptake of the carrier-containing drug by the CD133-expressing HT29 cell line in vitro. The use of nanoconjugates results in an enhanced cytotoxicity of the drug compared to the non-targeted self-assembled nanoconjugate.	[114]
Cisplatin and irinotecan encapsulated in poly(d,l-lactide-*co*-glycolide)-*co*-poly(ethylene glycol) (PLGA–PEG)-based nanoparticles directed toward prostate cancer cells overexpressing PSMA receptors, by using PSMA ligand-S,S-2-(3-[5-amino-1-carboxypentyl]-ureido)pentanedioic acid.	Selective endocytotic uptake and controlled release of drug, allowing complexes to act as cytotoxic agents. Both agents exhibited synergistic activities, resulting in elevated cell killing.	[115]
Nanoparticle system prepared with poly(DL-lactic acid) (PLA), poly(ethylene glycol)-block-poly(propylene glycol)-block-poly(ethylene glycol) (PEG–PPG–PEG), and irinotecan.	Enhanced antitumor effect against Sarcoma 180 solid tumor. Nanoparticles may exhibit cytotoxic potential in solid tumors, distant from the administration site.	[116]
PEGylated polyamidoamine (PAMAM) dendrimers containing SN-38 conjugated with peptides-BR2 and CyLoP1.	Formulation is much more cytotoxic in the murine colon carcinoma (C26) cell line compared to SN38 in its native form. Enhanced uptake of the drug by cells and higher cytotoxicity was observed in vivo for the formulation compared to SN-38 alone.	[117]
Hyaluronic Acid ChemoTransport (HyACT^®^)	Improved responsiveness in CD44 positive tumor cells. In addition, a combination of improved progression-free survival in metastatic colorectal cancer has been demonstrated, when compared to normal irinotecan monotherapy.	[119]
SN-38 conjugated to gold nanoparticles via oligonucleotides complementary to specific mRNAs unique to cancer cells of Ewing sarcoma.	The drug was efficiently delivered and selectively released in both in vitro and in vivo conditions.	[120]
Self-assemble poly(l-lysine)-b-poly(l-leucine) (PLys-b-PLeu) polymersomes.	The carriers did not exhibit any cytotoxic activity in tested cell lines (HEK, NIH3T3, and A549). Moreover, the loading of irinotecan into polymersomes resulted in similar antitumor activity in vitro to that observed for free drug.	[121]

## Abbreviations

5-FU	5-fluorouracil
ABC	ATP-binding cassette transporters
ADR	Adverse drug reactions
AKT	RAC-alpha serine/threonine-protein kinase
APAF-1	Apoptotic protease-activating factor 1
APC	7-ethyl-10-[4-N-(5-aminopentanoic acid)-1-piperidino] carbonyloxycamptothecin
ATM	Serine-protein kinase ATM
BAK	Bcl-2 homologous antagonist
BAX	Apoptosis regulator protein BAX
BCL10	B-cell lymphoma/leukemia 10
BCL-xL	Anti-apoptotic protein BCL-Xl
CD24	Signal transducer CD24
CDC2	Cyclin-dependent kinase 1
CDC25C	M-phase inducer phosphatase 3
CDK4	Cyclin dependent kinase 4
CES1/2	Carboxylesterase converting enzymes
CHK2	Serine/threonine-protein kinase CHK2
CPT-11	7-ethyl-10-[4-(1-piperidino)-1-piperidino]-carbonyloxycamptothecine
CRC	Colorectal cancer
DDR	DNA damage response
DSB	Double strand break
FAS	Tumor necrosis factor receptor superfamily member 6
GOs	Graphene oxides
hBChE	Butyrylcholinesterase
MAPK	Mitogen-activated serine/threonine protein kinase
MCL-1	Myeloid leukemia cell differentiation protein
MDM2	Mouse double minute 2 homolog
mTOR	Serine/threonine-protein kinase mTOR
NANOG	Homeobox protein NANOG
NOXA	Phorbol-12-myristate-13-acetate-induced protein 1
NPC	7-ethyl-10-(4-amino-1-piperidino] carbonyloxycamptothecin
NSCLC	Non-small cell lung cancer
OATP1B1	Human organic anion transporter
OS	Overall survival
P38	Mitogen-activated protein kinase P38
PAMAM	PEGylated polyamidoamine
PEG	Polyethylene glycol
PFS	Progression-free survival
PLGA–PEG	Poly(d,l-lactide-*co*-glycolide)-*co*-poly(ethylene glycol)
pRB	Retinoblastoma gene product
PSMA	Prostate-Specific Membrane Antigen
PTK	Protein tyrosine kinase
PUMA	p53 up-regulated modulator of apoptosis/ Bcl-2-binding component 3, isoforms ½
PVP	Polyvinylpyrrolidone
SCLC	Small cell lung cancer
SIPS	Stress-induced premature senescence
SNPs	Single-nucleotide polymorphisms
TMP	Thymidine monophosphate production
TOP	Topoisomerase
TP53	Cellular tumor antigen p53
UGT	Uridine 5′-diphospho-glucuronosyltransferase enzymes
β-CD	β-cyclodextrin
